# Vegetarian and Plant-Based Nutrition in Belgian Hospitals: A Cross-Sectional Study Revealing Gaps and Opportunities for Healthier Food Environments

**DOI:** 10.3390/nu18111654

**Published:** 2026-05-22

**Authors:** Evelien Mertens, Peter Deriemaeker, Tom Peeters, Katrien Van Beneden

**Affiliations:** 1 Research Centre for Preventive Health Innovation, Erasmushogeschool Brussel, 1090 Brussels, Belgium; tom.peeters@ehb.be; 2 Nutrition and Dietetics Program, EhB School of Health & Environment, Erasmushogeschool Brussel, 1090 Brussels, Belgium; peter.deriemaeker@ehb.be (P.D.); katrien.van.beneden@ehb.be (K.V.B.); 3 Department of Movement and Sport Sciences, Faculty of Physical Education and Physiotherapy, Vrije Universiteit Brussel, 1050 Brussels, Belgium

**Keywords:** plant-based, vegetarian, meal options, hospital, sustainable

## Abstract

**Background/Objective:** Transitioning towards plant-based dietary patterns is essential to improve health and reduce environmental impact. Hospitals represent a key setting to implement such dietary shifts, yet data on the availability of plant-based meals in healthcare institutions remain scarce. **Methods:** A cross-sectional survey was conducted across Dutch-speaking hospitals in Belgium to assess the meal plans and whether vegetarian or fully plant-based meal options were available for patients. Besides availability, the frequency and perceived barriers were assessed. Furthermore, the meal plans were analyzed to get an overview of the vegetarian and plant-based food options that were offered in different types of Belgian hospitals. **Results:** The availability of plant-based meal options was limited across hospitals. No meaningful differences were observed between general hospitals and other hospital types, including psychiatric, rehabilitation, and specialized hospitals. While plant-based fats and oils were widely available, key protein-rich plant foods such as legumes and minimally processed meat alternatives were rarely offered in all types of hospitals. Knowledge gaps among food service staff were observed, and structural barriers—including the need to accommodate diverse dietary requirements—were reported. **Conclusions:** Belgian hospitals currently underutilize the potential of vegetarian and plant-based nutrition to support patient health and sustainability goals. Strengthening institutional food environments by increasing the availability of nutritionally adequate plant-based meals represents a feasible and impactful strategy to align hospital practice with dietary guidelines and preventive healthcare priorities.

## 1. Introduction

The transition towards more plant-based dietary patterns is increasingly recognized as a key strategy to address both human and planetary health challenges. Diets rich in plant-based foods are associated with reduced risks of non-communicable diseases, including cardiovascular disease, type 2 diabetes, and certain cancers, while also lowering environmental impacts such as greenhouse gas emissions, land use, and water consumption [1,2,3,4,5,6,7,8,9,10]. Despite these well-documented benefits, current dietary patterns in Flanders remain imbalanced, with only 40% of protein intake derived from plant-based sources and 60% from animal-based sources [11,12]. This gap highlights the need for systemic changes to align dietary practices with both health and sustainability recommendations. Vegetarian dietary patterns generally exclude meat and fish but may include animal-derived products such as dairy products and eggs, depending on the specific dietary practice. In contrast, fully plant-based or vegan diets aim to avoid all foods of animal origin and primarily emphasize vegetables, fruits, legumes, whole grains, nuts, and seeds. Although these dietary patterns differ in the degree of restriction of animal products, both are increasingly promoted within the context of health promotion and environmental sustainability [13]. Although the prevalence of vegetarian and fully plant-based dietary patterns in Belgium remains relatively limited, interest in reducing meat consumption and increasing plant-based food intake has grown in recent years. Belgian studies have shown growing awareness of the health and environmental impact of meat consumption, particularly among younger populations and semi-vegetarian consumers [14].

Compared with diets high in animal-derived foods, dietary patterns emphasizing plant-based foods generally require fewer natural resources and are associated with lower greenhouse gas emissions, reduced land use, and lower water consumption [2,15]. In parallel, these dietary patterns have consistently been associated with favorable health outcomes, including lower risks of cardiovascular disease, type 2 diabetes, obesity, and premature mortality [3,4,16,17,18].

Several international and regional initiatives have emphasized the importance of increasing the consumption of plant-based foods. The EAT-Lancet Commission, the Sustainable Development Goals, and national policies such as the Flemish Green Deal Protein Shift advocate for a transition towards predominantly plant-based diets to support both public health and environmental sustainability [19]. In addition, there is growing recognition that preventive nutrition strategies could reduce the burden of non-communicable diseases and associated healthcare costs, which currently represent a substantial proportion of total healthcare expenditure [20,21,22,23]. In 2019, more than 70% of global deaths were attributed to non-communicable diseases, including cardiovascular disease, type 2 diabetes, obesity, and certain cancers, many of which are strongly influenced by dietary patterns and other lifestyle-related factors [24].

Hospitals represent a unique and influential setting in this context. As healthcare institutions, they play a central role in both treatment and prevention, whilst functioning as controlled food environments where dietary practices can be nudged. Providing nutritionally adequate and health-promoting meals is particularly important for hospitalized patients, who may be at risk of malnutrition, frailty, or disease-related complications [20,25,26]. Moreover, hospital food services have the potential to contribute to broader public health goals by aligning meal provision with evidence-based dietary guidelines.

However, existing evidence suggests that hospital food provision often falls short of these recommendations. Meals frequently include processed meats, energy-dense foods, and products high in saturated fats and added sugars, while offering insufficient amounts of fruits, vegetables, whole grains, and legumes [27]. At the same time, there is increasing societal interest in plant-based nutrition, reflected in the expansion of vegetarian and plant-based meal options in various institutional settings such as schools, workplaces, and restaurants [28]. In healthcare settings, several initiatives such as the Healthy Hospital Program and plant-based menu interventions in hospitals in the United States and Europe have demonstrated the feasibility and potential benefits of integrating plant-based meals into routine care [29,30,31,32].

Despite these developments, the implementation of plant-based meal options in hospitals remains limited and is often influenced by organizational, financial, and logistical constraints rather than health considerations [26,33]. Furthermore, little is known about the actual availability and characteristics of vegetarian and fully plant-based meal options in hospital settings, particularly in European healthcare systems. In Belgium, no previous research has systematically assessed the extent to which hospitals provide such options.

Therefore, the aim of this study was to assess the availability of vegetarian and fully plant-based meal options in Dutch-speaking Belgian hospitals, including both general hospitals and more specialized hospital settings such as psychiatric and rehabilitation hospitals. By identifying current practices and potential gaps, this study seeks to provide insights that can support the development of healthier and more sustainable hospital food environments.

## 2. Materials and Methods

### 2.1. Study Design and Hospital Selection

The availability of vegetarian and fully plant-based meal options in Dutch-speaking Belgian hospitals (*n* = 113) was assessed using a self-developed online questionnaire, as no validated questionnaire specifically addressing plant-based meal provision in Belgian hospital settings was identified in the literature. The study focused on Dutch-speaking hospitals in Belgium to ensure a more homogeneous study context regarding language, organizational structures, and hospital food service practices. This approach also facilitated consistent interpretation of the questionnaire and reduced potential variability related to regional differences within the Belgian healthcare system. A distinction was made between general hospitals (GHs) and other hospitals (OHs). General hospitals (GHs) are medical-specialist centers providing treatment and nursing care with overnight stays for a broad patient population rather than focusing on specific demographic groups or particular physical or psychological conditions. In contrast, OHs generally target more specific patient populations, such as psychiatric and rehabilitation patients.

### 2.2. Questionnaire Development

The questionnaire was developed by the research team based on the study objectives, existing literature on plant-based nutrition and hospital food environments, and the practical context of hospital meal provision. The introduction of the questionnaire explicitly stated that it should be completed by an individual with sufficient knowledge of the hospital’s meal provision. Response options were structured according to two criteria: (1) frequency of availability (ranging from once per week to daily) or (2) presence versus absence in offering vegetarian or fully plant-based choices. In addition, data was collected on hospital type, barriers or challenges in providing vegetarian and fully plant-based meal options, and food costs. The questionnaire also assessed the availability of vegetarian bread accompaniments and meal components commonly offered in Belgian hospital settings, including eggs, cheese products, plant-based spreads, and sweet toppings. The full questionnaire is available in the Supplementary Materials.

### 2.3. Data Collection and Ethical Approval

The digital survey was created using LimeSurvey 2.0 and was distributed via email between 21 February 2022 and 31 March 2022. Hospitals were identified through publicly available lists of Dutch-speaking Belgian hospitals. The invitation email was sent to the general hospital contact address with the explicit request to forward the questionnaire to a staff member with sufficient knowledge of the hospital’s meal provision and menu planning. In cases of non-response, reminder emails were sent twice, resulting in a total of three contact attempts per hospital.

The study was approved by the Ethics Committee of the University Hospital Brussels (B.U.N. 1432021000649).

### 2.4. Statistical Analyses

Statistical analyses were conducted using SPSS 28.0 (SPSS Inc., Chicago, IL, USA), with a significance level set at 0.05. Data normality was assessed using the Kolmogorov–Smirnov test. In the analysis, GHs were compared with OHs, which included four university hospitals, one GH with a university affiliation, four rehabilitation hospitals, 11 private hospitals, and one unspecified hospital. The frequency of meal provision was categorized as either ≤3 times per week (once, twice, or three times per week) or ≥4 times per week (four, five, or six times per week, or daily). This categorization was used to distinguish between lower and higher frequency availability within a typical week and to facilitate statistical comparisons between hospital types. In addition, offering plant-based meal options on four or more days per week may reflect a more structurally integrated approach to plant-based meal provision, which aligns more closely with current dietary recommendations promoting a shift towards predominantly plant-based dietary patterns [19]. Chi-square tests were used to examine the relationship between hospital type (GHs vs. OHs) and meal provision frequency (≤3 times per week vs. ≥4 times per week). Additionally, a Mann–Whitney U test was performed to assess differences in food costs between GHs and OHs, as the data were not normally distributed.

## 3. Results

### 3.1. Characteristics of Participating Hospitals and Meal Provision Practices

Of the 45 participating hospitals, 30 were GHs, including five university hospitals or an affiliation with a university. In total, 16 hospitals, including four rehabilitation hospitals, 11 private hospitals, and one unspecified hospital, were grouped in the analyses and categorized as other hospitals (OHs). In the 45 participating hospitals, meal planning and food provision were reported to involve one or more of the following professional profiles: kitchen manager (77.8%), head dietitian (64.4%), chef (55.6%), administrative staff (6.7%), assistant chef (4.4%), or catering company (2.2%). Only 22.2% of respondents responsible for hospital meal provision were able to correctly identify the components of a vegetarian diet, whereas 46.7% correctly indicated what constitutes a fully plant-based diet.

Knowledge of vegetarian and plant-based foods was considered the least challenging aspect of providing vegetarian and plant-based meal options, while the greatest barrier was the need to accommodate a wide variety of diets and nutritional patterns.

For the statistical analyses, GHs (53.3%) were compared with OHs (46.7%). No statistically significant difference was found in food cost per meal between GHs (mean ± SD: €4.91 ± 2.31) and OHs (mean ± SD: €3.73 ± 1.50) (*p* = 0.121). In addition, no statistically significant difference was observed in the reported demand for vegetarian and plant-based meals across hospitals (*p* = 0.055).

### 3.2. Availability of Vegetarian and Plant-Based Meals

The provision of plant-based sauces (available in 79.2% of GHs and 90.5% of OHs) and fully plant-based broth in soups (available in 79.2% of GHs and 95.2% of OHs) was not significantly associated with hospital type (*p* > 0.05). In hospitals with a GH designation, mashed potatoes were primarily prepared using ready-made mixes (37.5%, requiring only water to be added), whereas OHs more frequently used a combination of animal- and plant-based ingredients (47.6%).

The provision of fats did not vary according to the type of hospital (*p* > 0.05; Table 1). Analysis of the responses regarding meat alternatives in hot meals (Table 2) reveals that vegetable burgers (*p* = 0.027) and vegetarian/vegan burgers (*p* = 0.014) are offered more frequently in GHs. The availability of other meat alternatives did not depend on the type of hospital (*p* > 0.05).

### 3.3. Bread Accompaniments and Vegetarian Meal Components

Table 3 presents the availability of bread accompaniments and vegetarian meal components across hospital types. Jam (*p* = 0.024) and cheese spread (*p* = 0.027) are offered more frequently in general hospitals (GHs), while peanut butter or nut/seed spreads are more likely to be offered in other hospitals (OHs) (*p* = 0.025).

### 3.4. Plant-Based Alternatives and Food Products

Plant-based supplements (Table 4) are more commonly available in other hospitals (OHs) (*p* = 0.029). Regarding drinks, the supply was quite similar across both hospital types. For milk (100.0% availability in both types of hospitals), soy drink (83.3% in GHs, 85.7% in OHs), almond drink (0.0% in both hospital types), oat drink (0.0% in GHs vs. 14.3% in OHs), and rice drink (4.2% in GHs, 0.0% in OHs), no statistically significant difference was observed based on hospital type (*p* > 0.05). In terms of desserts and snacks, the availability of milk chocolate (29.2% in GHs vs. 28.6% in OHs), dark chocolate (16.7% in GHs vs. 14.3% in OHs), nuts (12.5% in GHs vs. 4.8% in OHs), pudding (100.0% in both types), rice pudding/porridge (91.7% in GHs vs. 90.5% in OHs), yoghurt (100.0% in both types), soy pudding (95.8% in GHs vs. 90.5% in OHs), pastries/cakes (95.8% in GHs vs. 85.7% in OHs), fresh fruit (100.0% in GHs vs. 95.2% in OHs), Biscoff biscuits (45.8% in GHs vs. 71.4% in OHs), and ice cream (54.2% in GHs vs. 57.1% in OHs), as well as freeze-dried fruit and sorbet (0.0% in both types), did not differ significantly based on hospital type (*p* > 0.05). However, the availability of soy yoghurt (58.3% in GHs vs. 85.7% in OHs; *p* = 0.043) and fruit in jars or preserves (83.3% in GHs vs. 52.4% in OHs; *p* = 0.025) was significantly influenced by hospital type.

## 4. Discussion

The present study provides the first systematic overview of the availability of vegetarian and fully plant-based meal options in Dutch-speaking hospitals in Belgium. The findings indicate that the provision of such meals remains limited and is not meaningfully associated with hospital type, suggesting that the current hospital food environment does not yet reflect the growing evidence base supporting plant-based nutrition nor existing dietary recommendations.

These findings align with international evidence showing that hospitals have been slow to integrate plant-based nutrition into routine food provision. For example, a recent study in the United Kingdom reported limited incorporation of plant-based protein sources in hospital menus despite national sustainability and nutrition targets [33]. Together, these results highlight a broader implementation gap between dietary guidelines and real-world practice in healthcare settings.

A key observation in this study is the low availability of nutritionally important plant-based protein sources, particularly legumes and minimally processed alternatives such as tofu and tempeh. This is noteworthy given the strong evidence supporting the role of legumes in improving cardiometabolic health and their central position in dietary guidelines such as the EAT-Lancet reference diet [18,19]. In Flanders, the intake of legumes is already substantially below recommended levels, averaging only 4 g/day compared to the suggested 75 g/day [11,19].

The limited availability of these foods in hospital settings may therefore represent a missed opportunity to improve patient health. Offering plant-based meat alternatives in a hospital setting may facilitate dietary transitions in institutional settings and health benefits with improvements in certain cardiovascular risk markers. However, their nutritional quality such as protein quality, saturated fat content, and salt levels should be monitored [34,35].

From a clinical perspective, this gap is particularly relevant. Hospitalized patients, especially older adults and those with chronic conditions, are at increased risk of malnutrition, sarcopenia, and frailty [36]. Emerging evidence indicates that plant-based protein sources can support muscle mass, strength, and physical functioning when appropriately incorporated into the diet [37,38,39,40,41,42,43]. Furthermore, dietary patterns rich in plant-based foods, such as the Mediterranean and DASH diets, have been associated with a lower risk of frailty and improved overall health outcomes [43,44,45,46,47,48,49,50]. These benefits are partly mediated by the high content of dietary fiber, antioxidants, unsaturated fatty acids, and other bioactive compounds, which contribute to reduced inflammation and improved metabolic health [8,15,50,51,52]. Importantly, recent evidence highlights that the health effects of plant-based diets depend strongly on their quality, with unhealthy plant-based diets being associated with increased cardiometabolic risk [53]. While healthy plant-based diets offer important health benefits, careful planning is required to ensure adequate intake of key nutrients such as vitamins A, B12, and D, key minerals including calcium, iodine, iron, phosphorus and zinc, long-chain polyunsaturated omega-3 fatty acids (eicosapentaenoic acid and docosahexaenoic acid) and essential amino acids, particularly in vulnerable populations [54].

At the same time, it is important to consider the nutritional quality of plant-based alternatives offered in hospital settings. In the present study, soy-based drinks were the most commonly available plant-based milk alternatives, which is favorable given their relatively high protein quality and amino acid profile [55]. However, many other plant-based drinks contain limited protein and may not be suitable replacements for dairy in nutritionally vulnerable populations [56]. In addition, the frequent availability of “accidentally vegan” snacks and desserts—which are often high in sugar and saturated fats— highlights that not all plant-based options are inherently healthy [57]. This emphasizes the importance of prioritizing minimally processed, nutrient-dense plant foods when implementing plant-based menus in clinical settings.

An important and novel finding of this study is the limited knowledge of plant-based diets among staff involved in hospital meal provision. Only a minority of respondents were able to correctly identify the components of vegetarian or fully plant-based diets. This suggests that barriers to implementation are not solely logistical or financial, but also educational. In addition, respondents identified the need to accommodate a wide variety of dietary patterns and nutritional requirements as the greatest challenge associated with offering more vegetarian and fully plant-based meal options. Improving the knowledge and competencies of healthcare and food service professionals may therefore be a key step in facilitating the transition towards healthier and more sustainable hospital food environments, as successful implementation of food-related interventions requires adequately trained staff and interdisciplinary collaboration.

At the institutional level, hospital food provision is often influenced by operational constraints, including budget considerations, procurement systems, and the need to accommodate diverse dietary requirements [26]. However, evidence suggests that plant-based dietary patterns can be cost-effective or even cost-saving. Studies have shown that plant-based meals may reduce food costs compared to animal-based diets, both in household and institutional settings [58,59,60]. Furthermore, increasing the availability of plant-based options may contribute to reducing healthcare costs by improving population health and lowering the burden of diet-related diseases [61]. Hospitals, as large-scale food purchasers, are uniquely positioned to influence supply chains and promote the availability of sustainable food products [26].

The findings of this study should be interpreted in the light of several limitations. First, the response rate was limited to approximately 40% of Dutch-speaking hospitals, which may affect the generalizability of the results. Second, the use of self-reported data may introduce reporting bias. Third, although the questionnaire explicitly requested completion by staff members with sufficient knowledge of hospital meal provision, the use of a self-administered survey may have resulted in variability in respondents’ expertise regarding all menu-related aspects. Last, although the questionnaire was developed based on the study objectives and existing literature, it was not formally validated, which may affect the reliability and reproducibility of certain findings. However, the study also has important strengths, including its novel focus and the provision of the first comprehensive overview of plant-based meal availability in Belgian hospitals.

Future research should focus on identifying effective strategies to improve the availability and quality of plant-based meals in hospital settings. This may include the development of evidence-based guidelines, staff training initiatives, and the involvement of dietitians in menu planning and evaluation. Consequently, further research is needed to assess patient acceptance, nutritional adequacy, and clinical outcomes associated with increased plant-based meal provision in hospitals.

## 5. Conclusions

In conclusion, this study demonstrates that the availability of vegetarian and fully plant-based meal options in Belgian hospitals remains limited and insufficiently aligned with current nutritional and sustainability recommendations. Despite the growing body of evidence supporting plant-based dietary patterns for the prevention and management of chronic diseases, their integration into hospital food environments appears to be underdeveloped. This gap represents a missed opportunity for healthcare institutions to actively contribute to both patient health and broader public health and environmental goals. Hospitals are uniquely positioned to model healthy dietary behaviors and to support the implementation of evidence-based nutrition strategies within clinical care.

Addressing this gap will require a multifaceted approach, including improving the availability and nutritional quality of plant-based meals, strengthening knowledge and competencies among healthcare and food service professionals, and embedding plant-based nutrition within institutional policies and procurement practices. Advancing plant-based nutrition in hospital settings has the potential to contribute meaningfully to improved patient outcomes, reduced healthcare costs, and more sustainable food systems.

## Figures and Tables

**Table 1:** Availability of fats/oils (baking/roasting and spreading) in general hospitals (GHs) and other hospitals (OHs).

	GHs		OHs		
Fats (Baking and Roasting)	≤3d/w	≥4d/w	≤3d/w	≥4d/w	*p*-Value
Animal butter and/or ghee	54.2%	45.8%	76.2%	23.8%	0.124
Vegetable margarine (packed in tub/tray)	8.3%	91.7%	4.8%	95.2%	0.632
Vegetable oil (e.g., olive oil, sunflower oil, …)	62.5%	37.5%	52.4%	47.6%	0.493
Mix of vegetable and animal fat	75.0%	25.0%	85.7%	14.3%	0.370
**Fats (spreading)**					
100% vegetable margarine (packed in tub/tray)	8.3%	91.7%	4.8%	95.2%	0.632
Mix of vegetable and animal fat	75.0%	25.0%	81.0%	19.0%	0.632
Animal butter (wrapped in wrapper)	62.5%	37.5%	85.7%	14.3%	0.079

**Table 2:** Availability of meat alternatives in hot meals in general hospitals (GHs) and other hospitals (OHs).

	GHs		OHs		
	≤3d/w	≥4d/w	≤3d/w	≥4d/w	*p*-Value
Chickpeas	100.0%	0.0%	95.2%	4.8%	0.280
Split peas	100.0%	0.0%	100.0%	0.0%	/
Lentils	100.0%	0.0%	100.0%	0.0%	/
White/black/brown/kidney/lupine/borlotti beans *	100.0%	0.0%	100.0%	0.0%	/
Nuts	95.8%	4.2%	100.0%	0.0%	0.344
Eggs	75.0%	25.0%	90.5%	9.5%	0.176
Tofu	91.7%	8.3%	100.0%	0.0%	0.176
Seitan	100.0%	0.0%	100.0%	0.0%	/
Tempeh	100.0%	0.0%	100.0%	0.0%	/
Falafel	95.8%	4.2%	100.0%	0.0%	0.344
Mycoprotein (for example: Quorn©)	75.0%	25.0%	81.0%	19.0%	0.632
Vegetable burger	79.2%	20.8%	100.0%	0.0%	**0.027**
Vegetarian/vegan minced meat	91.7%	8.3%	100.0%	0.0%	0.176
Vegetarian/vegan meatballs	87.5%	12.5%	100.0%	0.0%	0.094
Vegetarian/vegan burger	75.0%	25.0%	100.0%	0.0%	**0.014**
Vegetarian/vegan schnitzel	91.7%	8.3%	100.0%	0.0%	0.176
Vegetarian/vegan strips	100.0%	0.0%	100.0%	0.0%	/

* These were all questioned separately in the questionnaire, but all showed the same result.

**Table 3:** Availability of bread accompaniments and vegetarian meal components in general hospitals (GHs) and other hospitals (OHs).

	GHs		OHs		
	≤3d/w	≥4d/w	≤3d/w	≥4d/w	*p*-Value
Jam	4.2%	95.8%	28.6%	71.4%	**0.024**
Chocolate spread	37.5%	62.5%	42.9%	57.1%	0.714
Speculoos paste	45.8%	54.2%	52.4%	47.6%	0.661
Peanut butter or nut/seed spread	100.0%	0.0%	81.0%	19.0%	**0.025**
Hummus	83.3%	16.7%	66.7%	33.3%	0.194
Lentil spread	100.0%	0.0%	90.5%	9.5%	0.122
Eggs	70.8%	29.2%	81.0%	19.0%	0.431
Slices of cheese (non-vegetarian = animal curd)	25.0%	75.0%	33.3%	66.7%	0.538
Slices of cheese (vegetarian = non-animal curd)	75.0%	25.0%	81.0%	19.0%	0.632
Fresh cheese and cottage cheese	20.8%	79.2%	47.6%	52.4%	0.057
Cheese spread (e.g., La Vache Qui Rit, …)	20.8%	79.2%	52.4%	47.6%	**0.027**
Soft cheese (e.g., brie, camembert, …)	62.5%	37.5%	71.4%	28.6%	0.526
Vegetarian/vegan slices	79.2%	20.8%	57.1%	42.9%	0.111

**Table 4:** Availability of plant-based supplements in general hospitals (GHs) and other hospitals (OHs).

	GHs			OHs			
	1	2	3	1	2	3	*p*-Value
Plant-based supplements (=plant-based types of Fresubin, Fortimel, etc.)	50.0%	50.0%	25.0%	81.0%	19.0%	0.0%	**0.029**
*1: Yes, this is standardly available*							
*2: No, this is not standard, but can be ordered and delivered quickly*							
*3: No, it is not possible to order plant-based supplements for patients*							

## Data Availability

The data presented in this study are available in Table 1, Table 2, Table 3 and Table 4.

## Supplementary Materials

The following supporting information can be downloaded at: https://www.mdpi.com/article/10.3390/nu18111654/s1. English translation of the questionnaire used to assess vegetarian and fully plant-based meal provision in Belgian hospitals.
