# Supplementary material for: Vegetarian and Plant-Based Nutrition in Belgian Hospitals: A Cross-Sectional Study Revealing Gaps and Opportunities for Healthier Food Environments
Source: Nutrients. 2026 May 22;18(11):1654. doi: 10.3390/nu18111654 (PMC13258568; doi:10.3390/nu18111654)
Supplement: Supplementary file 1 [file nutrients-18-01654-s001.zip › nutrients-4316853-supplementary.pdf]

Supplementary Material S1

English translation of the questionnaire used to assess vegetarian and fully plant-based meal provision in Belgian hospitals

Questionnaire

1. What type of hospital do you work in? (one answer possible)

- General hospital
- University hospital
- General hospital with university affiliation
- Rehabilitation hospital
- Private hospital
- Other: ______

2. Who is responsible for patient menu planning in the hospital? (multiple answers possible)

- Head dietitian
- Kitchen manager
- Chef
- Assistant chef
- Catering company (e.g., Sodexo, ISS)
- Administrative staff
- Other: ______

3. Does the hospital have a cafeteria or restaurant for staff and visitors? (one answer possible)

- Yes
- No

4. Who is responsible for menu planning for staff and visitors in the cafeteria/restaurant? (multiple answers possible)

- Head dietitian
- Kitchen manager
- Chef
- Assistant chef
- Catering company (e.g., Sodexo, ISS)
- Administrative staff
- Other: ______

5. Which meals are offered daily in the hospital? (multiple answers possible)

- Breakfast
- Hot lunch meal
- Evening bread meal
- Snacks

6. Is there frequent demand from patients for vegetarian meals? (one answer possible)

- Yes, regularly
- No, not really
- I do not know

7. What is the average food cost of the three daily meals?

- _____ euro

8. Approximately how many kitchen staff members follow a vegetarian or vegan diet?

- _____ persons

9. How does the kitchen staff generally perceive vegetarian and vegan meals? (1 = very negative; 5 = very positive)

- 1 – 2 – 3 – 4 – 5

10. What do you understand by a vegetarian dietary pattern? A vegetarian dietary pattern excludes: (multiple answers possible)

- Meat
- Fish
- Shellfish and crustaceans
- Eggs
- Milk
- Dairy products
- Cheese
- Honey
- Gelatin

11. What do you understand by a vegan dietary pattern? A vegan dietary pattern excludes: (multiple answers possible)

- Meat
- Fish
- Shellfish and crustaceans
- Eggs
- Milk
- Dairy products

- Cheese
- Honey
- Gelatin

12. How can patients indicate which dietary pattern or specific diet they follow? (multiple answers possible)

- Via the dietitian(s)
- Via nursing staff
- Via physicians
- Via a logistics staff member visiting the patient room
- Other: ______

13. Which barriers or challenges does the hospital experience when offering vegetarian or vegan meals? (open-ended question)

14. To what extent are the following perceived as barriers when offering vegetarian or vegan meals to patients? (1 = very small barrier; 5 = very large barrier)

- Financial: food cost of vegetarian and vegan meals
- Knowledge of vegetarian and vegan nutrition
- Inspiration for vegetarian and vegan recipes
- Practical/technical: production lines
- Practical: accommodating multiple dietary patterns in the hospital
- Training of kitchen staff

15. To what extent are the following perceived as barriers when offering vegetarian or vegan meals in the cafeteria for staff and visitors? (1 = very small barrier; 5 = very large barrier)

- Financial: food cost of vegetarian and vegan meals
- Knowledge of vegetarian and vegan nutrition
- Inspiration for vegetarian and vegan recipes
- Practical/technical: production lines
- Practical: accommodating multiple dietary patterns in the hospital

16. How often are the following fats or cooking fats used during bread meals for patients?

- Animal butter and/or ghee
- Plant-based margarine
- Vegetable oil (e.g., olive oil, sunflower oil)
- Combination of plant-based and animal fats
- Other: ______

17. How often are the following fats or cooking fats used during bread meals in the cafeteria for staff and visitors?

- Animal butter and/or ghee
- Plant-based margarine
- Vegetable oil (e.g., olive oil, sunflower oil)
- Combination of plant-based and animal fats
- Other: ______

18. How often are the following foods offered as part of hot meals for patients?

- Chickpeas
- Split peas
- Lentils
- Beans (white, black, brown, kidney, lupine, borlotti)
- Tofu
- Seitan
- Tempeh
- Eggs
- Falafel
- Mycoprotein (e.g., Quorn®)
- Vegetable burger
- Vegetarian/vegan minced meat
- Vegetarian/vegan meatballs
- Vegetarian/vegan burger
- Vegetarian/vegan schnitzel
- Vegetarian/vegan strips
- Nuts
- Other: ______

19. How often are the following foods offered as part of hot meals in the cafeteria for staff and visitors?

- Chickpeas
- Split peas
- Lentils
- Beans (white, black, brown, kidney, lupine, borlotti)
- Tofu
- Seitan
- Tempeh
- Eggs
- Falafel
- Mycoprotein (e.g., Quorn®)
- Vegetable burger

- Vegetarian/vegan minced meat
- Vegetarian/vegan meatballs
- Vegetarian/vegan burger
- Vegetarian/vegan schnitzel
- Vegetarian/vegan strips
- Nuts
- Other: ______

20. How often are the following spreads or fats offered during bread meals for patients?

- 100% plant-based margarine
- Combination of plant-based and animal fats
- Animal butter
- Other: ______

21. How often are the following spreads or fats offered during bread meals in the cafeteria for staff and visitors?

- 100% plant-based margarine
- Combination of plant-based and animal fats
- Animal butter
- Other: ______

22. How often are the following bread accompaniments or meal components offered during bread meals for patients?

- Jam
- Chocolate spread
- Speculoos spread (cookie butter)
- Peanut butter or nut/seed spread
- Hummus
- Lentil spread
- Eggs
- Cheese slices (non-vegetarian = animal rennet)
- Cheese slices (vegetarian = non-animal rennet)
- Fresh cheese and cottage cheese
- Cheese spread
- Soft cheese (e.g., brie, camembert)
- Vegetarian/vegan sandwich slices
- Other: ______

23. How often are the following bread accompaniments or meal components offered during bread meals in the cafeteria for staff and visitors?

- Jam
- Chocolate spread
- Speculoos spread (cookie butter)
- Peanut butter or nut/seed spread
- Hummus
- Lentil spread
- Eggs
- Cheese slices (non-vegetarian = animal rennet)
- Cheese slices (vegetarian = non-animal rennet)
- Fresh cheese and cottage cheese
- Cheese spread
- Soft cheese (e.g., brie, camembert)
- Vegetarian/vegan sandwich slices
- Other: ______

24. Which milk options can patients choose for coffee? (multiple answers possible)

- Skimmed/semi-skimmed/full-fat milk
- Soy drink
- Almond drink
- Oat drink
- Rice drink
- Other: ______

25. Which milk options can staff and visitors choose for coffee in the cafeteria? (multiple answers possible)

- Skimmed/semi-skimmed/full-fat milk
- Soy drink
- Almond drink
- Oat drink
- Rice drink
- Other: ______

26. Which desserts/snacks are available for patients? (multiple answers possible)

- Speculoos biscuit
- Milk chocolate
- Dark chocolate
- Nuts
- Pudding
- Rice pudding
- Yogurt
- Soy yogurt
- Soy pudding

- Pastries/cakes (e.g., waffles, cake)
- Fresh fruit
- Fruit in jars or canned fruit
- Dried/freeze-dried fruit
- Ice cream
- Sorbet
- Other: ______

27. Which desserts/snacks are available for staff and visitors in the cafeteria? (multiple answers possible)

- Speculoos biscuit
- Milk chocolate
- Dark chocolate
- Nuts
- Pudding
- Rice pudding
- Yogurt
- Soy yogurt
- Soy pudding
- Pastries/cakes (e.g., waffles, cake)
- Fresh fruit
- Fruit in jars or canned fruit
- Dried/freeze-dried fruit
- Ice cream
- Sorbet
- Other: ______

28. Which type of broth is used for soup preparation? (multiple answers possible)

- Vegetable broth (not fully plant-based)
- Fully plant-based vegetable broth
- Beef broth
- Chicken broth
- Other: ______

29. How is mashed potato typically prepared? (multiple answers possible)

- Ready-made mix (only water added)
- With animal-derived ingredients (e.g., egg, butter, milk)
- With plant-based ingredients (e.g., plant-based margarine/oil, unsweetened soy drink)
- Combination of animal-derived and plant-based ingredients
- Other: ______

30. How often are sauces (e.g., meat sauce, cheese sauce, béchamel sauce) offered with hot meals for patients?

- Response options: Never, 1x/week, 2x/week, 3x/week, 4x/week, 5x/week, 6x/week, Daily.

31. How often are sauces (e.g., meat sauce, cheese sauce, béchamel sauce) offered with hot meals in the cafeteria for staff and visitors?

- Response options: Never, 1x/week, 2x/week, 3x/week, 4x/week, 5x/week, 6x/week, Daily.

32. Is it possible to prepare fully plant-based sauces for patients?

- Yes
- No

33. Is it possible to prepare fully plant-based sauces for staff and visitors in the cafeteria?

- Yes
- No

34. Are plant-based oral nutritional supplements (e.g., plant-based versions of Fresubin®, Fortimel®) available for patients?

- Yes, these are standardly available
- No, these are not standardly available but can be ordered quickly
- No, it is not possible to order plant-based oral nutritional supplements
